# Return to work after specialized rehabilitation—An explorative longitudinal study in a cohort of severely disabled persons with stroke in seven countries: The Sunnaas International Network stroke study

**DOI:** 10.1002/brb3.1055

**Published:** 2018-07-18

**Authors:** Birgitta Langhammer, Katharina S. Sunnerhagen, Susanne Sällström, Frank Becker, Johan K. Stanghelle

**Affiliations:** ^1^ Faculty of Health Sciences Sunnaas Rehabilitation Hospital Oslo Metropolitan University Oslo Norway; ^2^ Sunnaas Rehabilitation Hospital Nesoddtangen Norway; ^3^ Department of Clinical Neuroscience Faculty of Medicine University of Gothenburg Göteborg Sweden; ^4^ Faculty of Medicine University of Oslo Nesoddtangen Norway

**Keywords:** disability, rehabilitation, return to work, stroke

## Abstract

**Introduction:**

Stroke may impose disabilities with severe consequences for the individual, with physical, psychological, social, and work‐related consequences. The objective with the current study was to investigate to what extent persons with stroke were able to return to work, to maintain their financial situation, and to describe the follow‐up services and participation in social networks and recreational activities.

**Methods:**

The design was a prospective, descriptive study of specialized stroke rehabilitation in nine rehabilitation centers in seven countries. Semistructured interviews, which focused on the return to work, the financial situation, follow‐up services, the maintenance of recreational activities, and networks, were performed 6 and 12 months post discharge from rehabilitation.

**Results:**

The working rate before the onset of stroke ranged from 27% to 86%. At 12 months post stroke, the return to work varied from 11% to 43%. Consequently, many reported a reduced financial situation from 10% to 70% at 6 months and from 10% to 80% at 12 months. Access to postrehabilitation follow‐up services varied in the different countries from 24% to 100% at 6 months and from 21% to 100% at 12 months. Physical therapy was the most common follow‐up services reported. Persons with stroke were less active in recreational activities and experienced reduced social networks. Associations between results from the semistructured interviews and related themes in LiSat‐11 were small to moderate. The study shows that education, age, and disability are predictors for return to work. Differences between countries were observed in the extent of unemployment.

**Conclusions:**

In this international multicentre study, return to work after severe stroke and specialized/comprehensive rehabilitation was possible, depending on the extent of the disability, age, and education. Altered financial situation, reduced social networks, and reduced satisfaction with life were common psychosocial situations for these patients.

## 1 INTRODUCTION

It is estimated that 15 million people worldwide survive strokes each year (Feigin et al., 2014). Stroke may impose disabilities with severe consequences for the individual, not only in the physical and psychological sense but also with regard to the patient's social situation, work, and family relations. Return to work (RTW) has been shown to improve their overall life satisfaction as well as economic circumstances (Bonner et al., 2016; Treger, Shames, Giaquinto, & Ring, 2007; Vestling, Tufvesson, & Iwarsson, 2003). Factors influencing the RTW after stroke have been related to the financial and intrinsic aspects of the work and focused less on its social aspects (Vestling et al., 2003). There seems to be a reciprocal relationship between RTW and life satisfaction. RTW seems to strengthen overall subjective life satisfaction (Vestling et al., 2003). Social consequences, if a RTW is impossible, have been reported: such as a negative impact on family relationships, a deterioration in sexual life, economic difficulties, and the curtailment of leisure activities (Daniel, Wolfe, Busch, & McKevitt, 2009). It is known that a better functional outcome 1‐year post stroke has been associated with a return to paid work (Busch, Coshall, Saka, & Wolfe, 2007). However, it is also reported that 53% of 161 persons who are independent according to the Barthel Index (BI > 19) and 39% of 96 persons who are very active according to the Frenchay Activities Index (FAI > 30/45) did not resume work (Busch et al., 2007; Sæki, 2000; Saeki, Matsushima, Kato, Itoh, & Shiraishi, 2016). These results indicate that working status depends on other explanatory factors than independence and activity levels, which often are the primary outcomes in rehabilitation (Busch et al., 2007; Sæki, 2000; Saeki et al., 2016). Therefore, despite excellent functional outcome post stroke after rehabilitation, a large proportion of persons with stroke do not resume work, which may have economic consequences for the individual and for the family—as well as for society. In addition, one may speculate that RTW influences ability to maintain a social life with networks and self‐esteem, especially in cultures where individual independence is a vital virtue (Steele & Lynch, 2013). Work and return to work may also be viewed as a social arena related to financial independence, contacts with social network and as such vital for self‐esteem, that is the confidence in one's own worth or abilities (Vestling et al., 2003).

There are few, if any, studies focusing on stroke patients’ social situation in persons with moderate to severe stroke and even less in a multinational setting. Sunnaas International Network has initiated and performed a multinational research project to investigate the content of specialized rehabilitation, outcomes, and social consequences after stroke during and after specialized rehabilitation in nine clinics situated in seven countries (Langhammer et al., 2015; Langhammer, Sunnerhagen, Lundgren‐Nilsson, et al., 2017; Langhammer, Sunnerhagen, Stanghelle, et al., 2017; Stanghelle et al., 2015). The British Society of Rehabilitation Medicine (BSRM) as has defined specialized rehabilitation: “services that support patients with complex disability, whose rehabilitation needs are beyond the scope of their local rehabilitation services” (BSRM, 2018).

At 6 and 12 months post discharge from rehabilitation, semistructured interviews were conducted, in which the participants were asked to describe the major changes in their lives with regard to the job situation, the financial situation, the social situation, the follow‐up services from society, the living situation, and medical status following rehabilitation after the stroke.

The aims of this international multicentre study were as follows:

- To investigate to what extent persons with moderate to severe stroke were able to RTW and to maintain their financial situation in the first year after stroke rehabilitation.
- To describe the extent of follow‐up services and to what extent networks and recreational activities were maintained 6 and 12 months post rehabilitation.
- To explore how the RTW influenced maintenance of social networks and self‐esteem.

The hypotheses were that their physical ability and level of independence would positively influence working status and life satisfaction and that the RTW and maintenance of recreational activities were related to the maintenance of social networks and self‐esteem.

### 1.1 Ethics

Approvals of the local ethical committees in each country were obtained in 2012, including that of the Regional Ethics Committee of Health in Norway (2012/768). Information on the aim of the study was given to the participants both verbally and in writing, and written informed consent was obtained. The study was registered in ClinicalTrialsGov: NCT01732679.

## 2 MATERIAL AND METHODS

### 2.1 Design

The design was a prospective, descriptive study of specialized rehabilitation for persons with stroke, at nine clinics in seven countries: Sunnaas Rehabilitation Hospital, Norway; China Rehabilitation and Research Center (CRRC) and Bayi, China; Rusk Institute, United States; Policlinica 2, Russia; El Wafa, Gaza; Bethlehem Arab Society of Rehabilitation (BASR), Palestine; Sheba Medical Hospital, Israel; and Högsbo Rehabilitation Hospital, Sweden. Persons with a primary diagnosis of stroke, as defined by the World Health Organization (WHO) were consecutively enrolled in the study (Langhammer et al., 2015). Inclusion criteria were that the patients had a severe first‐time stroke, defined as a modified Rankin Scale (mRS) score 3–4, in need of specialized rehabilitation post stroke and cognitively able to understand the objectives of the study and participated voluntarily. Patients with subarachnoid hemorrhage, malignancy, or other severe medical conditions in combination with stroke were excluded.

Patients were followed up at 6 and 12 months post discharge from rehabilitation. The goal was to include 30 patients from each clinic in a descriptive explorative design of standard treatment. Data were collected from September 2012 to September 2014.

### 2.2 Outcome measures

#### 2.2.1 Descriptive

Descriptive data of the patients with stroke admitted for inpatient rehabilitation to the participating hospitals were obtained to highlight stroke criteria, severity and disability post stroke, other severe medical conditions, medication, the time between onset and admission, and length of stay (LOS).

The degree of disability and the severity of the stroke were evaluated with the National Institute of Health Stroke Scale (NIHSS; Meyer, Hemmen, Jackson, & Lyden, 2002) and the modified Rankin Scale (mRS; Rankin, 1957) on admission to the inpatient rehabilitation unit. In addition, the Barthel Index (BI; Mahoney & Barthel, 1965), alternatively the Functional Independence Measure (FIM; Hsueh, Lin, Jeng, & Hsieh, 2002), for performance in ADL (Hsueh et al., 2002; Mahoney & Barthel, 1965), and LiSat‐11, perceived satisfaction with life with the Life Satisfaction Checklist (LiSat‐11; Fugl‐Meyer, Melin, & Fugl‐Meyer, 2002; Viitanen, Fugl‐Meyer, Bernspång, & Fugl‐Meyer, 1988), were applied on admittance, 18–22 days into rehabilitation, at discharge, and at 6 and 12 months after discharge.

Semistructured interviews were conducted at 6 and 12 months post discharge from rehabilitation.

### 2.3 Main outcome measure

A study‐specific medical file including semistructured interviews with the focus on RTW, the financial situation, follow‐up services, maintenance of recreational activities, and networks was the main outcome measure performed 6 and 12 months post discharge in each of the clinics by an independent tester.

The questions were categorized into three alternative answers yes/no/not applicable, for questions about work, finance, follow‐up services, recreational activities, and social networks with the possibility of adding alternatives such as “as before, more or less.” The questions referred to present time at the 6 and 12 months post discharge. The last question in the semistructured interview was answered narratively: “Please briefly describe the major change in your life after the incidence of stroke.”

*The National Institute of Health Stroke Scale (NIHSS)* is a stroke‐specific quantitative scale (Meyer et al., 2002). NIHSS scores reflect the severity of neurological deficits with a 0 score implying no deficits, a score of 1–4 indicating a minor stroke, 5–15 a moderate stroke, 16–20 moderate to severe stroke, and 21–42 a severe stroke.

*The modified Rankin Scale (mRS)* defines six levels of disability (0–5) and one for death (Busch et al., 2007; Rankin, 1957). A score of zero indicates no symptom at all, the higher scores indicate a more severe disability, and the score of 6 indicates death.

*Activities of Daily Living (ADL)—*The BI is a test of the primary activities of daily living developed by Mahoney and Barthel (Mahoney & Barthel, 1965) and the FIM measures the patients’ performance in the basic activities of daily living consisting of 18 items, of which the 13 motor tasks (FIM‐M) were performed. Subsequent studies have demonstrated that the psychometric properties of FIM and BI are similar (Hsueh et al., 2002). A dichotomization of FIM‐M scores to lower (0–55) and higher scores (56–91) has been used, comparable to BI 0–59 and 60–100 (Kwon, Hartzema, Duncan, & Min‐Lai, 2004). The lower scores were categorized as dependent and the higher scores as partially independent. Correlations between BI and FIM‐M have been established as 0.95, *p* < 0.0001 (Kwon et al., 2004). FIM‐M was transformed from 91 to 100 scales by inserting raw FIM‐M scores into: x/91 × 100 for both test occasions for analysis and comparisons with the BI total score and named ADL.

*LiSat‐11—*Perceived satisfaction with life was evaluated according to the generic Life Satisfaction Checklist (LiSat‐11; Fugl‐Meyer et al., 2002; Viitanen et al., 1988). The LiSat‐11 entails one global item and 10 domain‐specific items capturing life satisfaction, rated on a six‐step ordinal scale ranging from very dissatisfying (=1) to very satisfying (=6).

### 2.4 Data analysis

Statistical analyses were performed in IBM SPSS statistics version 24.0, http://scicrunch.org/reslover/SCR_002865. Demographics at baseline are presented in mean and standard deviations. Descriptive data of the psychosocial situation are presented in frequencies and percentages. Non‐parametric paired Wilcoxon signed rank test were performed to establish possible differences for the interview questions between 6 and 12 months. This was done to establish whether there were significant differences between the two time points in any of the questions and to what extent these differences were general or specific to clinics.

To evaluate predictors for the RTW and financial situation, a logistic regression analysis was performed, with employment status categorized as employed/unemployed entered as dependent and age, gender, education, country, NIHSS, mRS, and ADL as independent variables. The significance level was set at *p* < 0.05 (Altman, 2001).

Using comparative text analysis, the semistructured questionnaires were analyzed qualitatively, with an inductive approach, to extract the main categories of perceived major changes (Malterud, 2012). Two of the co‐authors (BL, SS) performed the analyses in four stages: reading, interpreting, synthesizing into categories, and reaching consensus (Malterud, 2012).

## 3 RESULTS

In total, 230 persons with severe stroke were enrolled consecutively (Table 1). At baseline, that is, on admission to rehabilitation, on average 53.4% of the stroke patients had been working, 29.1% were retired, and 17.4% were unemployed. The employment status among participants varied from 27% to 86% between the clinics.

**Table 1:** Descriptives at baseline in the different settings: Sunnaas Rehabilitation Hospital, Norway; China Rehabilitation Research Center, China; Rusk Institute, United States; Policlinica 2, Russia; Sheba Medical, Israel; Bethlehem Arab Society Rehabilitation, Palestine; El Wafa, Gaza; Högsbo‐Sahlgrenska University Hospital, Sweden; and Bayi, China

	Sunnaas (*n *= 31)	CRRC (*n *= 35)	Rusk Inst (*n *= 17)	Pol. clin 2 (*n *= 30)	Sheba (*n *= 14)	BASR (*n *= 30)	El Wafa (*n *= 8)	SU/Högsbo (*n *= 30)	Bayi (*n *= 35)
Age, years (m, *SD*)	59.7 (9.8)	49.5 (13.5)	63.6 (16.1)	58.2 (13.4)	61.2 (12.6)	60.2 (14.4)	58.1 (11.9)	57.3 (5.2)	58.5 (14.1)
Gender (male/female)	22/9	23/12	9/8	18/12	12/2	16/14	5/3	27/3	22/13
Marital status: married/single/widow	14/17/0	31/4/0	9/6/2	23/2/5	12/1/1	21/2/7	7/0/1	19/11/0	31/2/2
Years in education									
0–6 years/7–12 years/13+	1/14/16	3/17/15	0/3/14	1/9/20	1/5/8	16/9/5	1/2/5	0/19/11	9/15/11
NIHSS^a^	7.5	6.5	4.4	11.7	6.4	8.4	6.1	6.0	9.9
mRS^b^	3.7	3.6	4.1	3.3	4.4	3.7	3.3	3.4	4.1
ADL (0–100; m, *SD*)	67.5 (17.2)	45.6 (22.6)	49.6 (17.5)	56.7 (25.4)	43.9 (27.1)	45.9 (17.8)	49.4 (29.1)	64.8 (17.1)	38.9 (26.0)
Length of stay (LOS; m, *SD*)	43.3 (25.6)	69.3 (37.9)	21.9 (5.8)	51.3 (9.1)	82.5 (36.7)	34.8 (22.3)	33.9 (26.1)	46.1 (16.1)	59.3 (35.8)

a 0 = no stroke symptoms, 1–4 = minor, 5–15 = moderate, 16–20 = moderate to severe, 21–42 = severe symptoms.

b 1 = no stroke symptoms, 2 = minor, 3 = moderate, 4 = moderate to severe, 5 = severe symptoms, 6 = dead.

The overall baseline disability rate was a mean of 3.7 (*SD* 0.8)/median 4 (IQR 1.0) modified Rankin Scale (mRS), and for severity by mean 7.8 (*SD* 4.4)/median 7 (IQR 6.6), National Institute of Health Stroke Scale (NIHSS). Persons enrolled were younger than the general population of stroke victims with more males than females (Table 1).

At 6 and 12 months post discharge, 200 and 182 patients, respectively, were eligible for the interviews. In total, at 6 and 12 months, 18%/20% had been able to RTW, 42%/33% had not been able to RTW, and 28%/45% were retired by that time. The numbers indicate a majority of nonworkers in the follow‐up period. In each clinic, the RTW varied from 7% to 43% at 6 months and from 10% to 43% at 12 months, indicating a slight increase in employment, with two exceptions El Wafa, Gaza, and Bayi, China (Table 2). Vocational status was significantly different between clinics at baseline, but not at 6 and 12 months.

**Table 2:** Persons with stroke on admission, 6 and 12 months post discharge (*n* = x/x/x) who reported having returned to work, in percentages, in the nine clinician the SIN network

	Sunnaas (*n* = 31/24/18)	CRRC (*n* = 35/28/28)	Rusk (*n* = 17/14/12)	Pol. clin 2 (*n* = 30/30/30)	Sheba (*n* = 14/12/8)	BASR (*n* = 30/22/21)	El Wafa (*n* = 8/8/8)	SU/Högsbo (*n* = 30/28/27)	Bayi (*n* = 35/34/29)	
Status (%) empl/unempl/ret^a^										
Baseline	52/19/29	63/9/29	59/6/35	53/10/37	86/7/7	27/53/20	50/25/25	77/10/13	31/17/51	0.005*
6 months post discharge	7/32/36	14/26/40	30/30/30	43/23/33	7/79/7	7/63/3	38/13/50	13/70/10	17/37/43	0.15
12 months post discharge	42/13/45	17/32/52	35/12/41	43/23/33	21/36/43	10/53/37	25/25/50	20/53/23	11/31/54	0.14

a Employed/unemployed/retired.

*p < 0.05.

**Figure 1:**
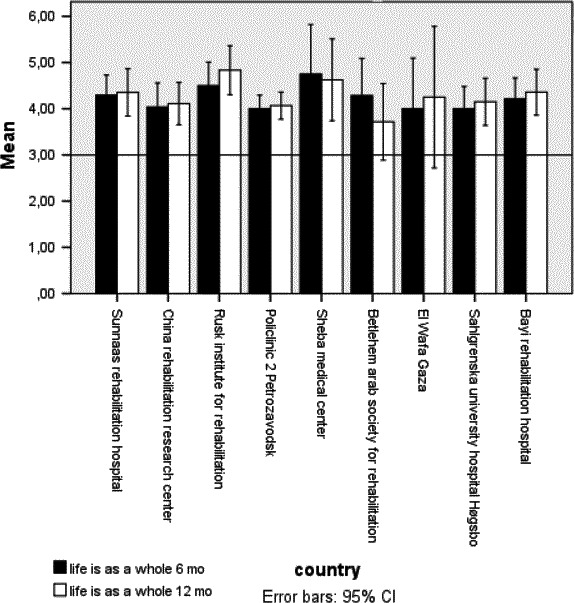
Satisfaction with life as a whole 6 and 12 months post stroke rehabilitation

**Figure 2:**
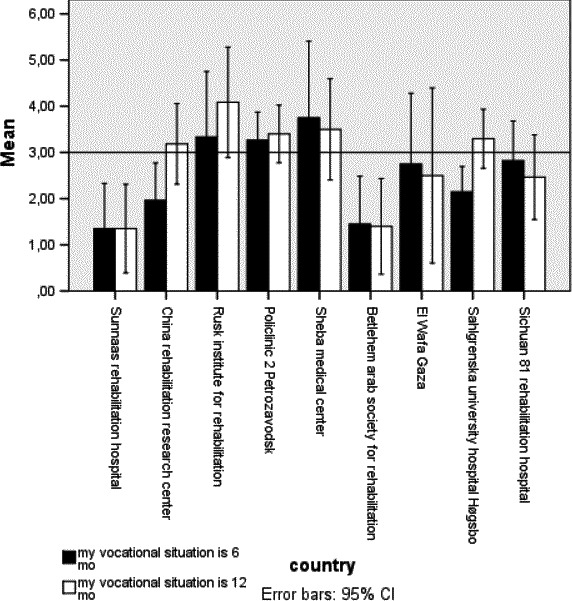
Satisfaction with vocational situation 6 and 12 months post stroke rehabilitation

Satisfaction with score items “life as a whole” in general (Langhammer et al., 2017) and the “vocational situation” in LiSat‐11 was reported to be low in the majority of clinics (Figures 1 and 2). There was a low but significant association between RTW and satisfaction with the vocational situation at 6 and 12 months (rho = −0.31 and rho = −0.29).

Regarding the participants’ perception of work, financial situation, follow‐up services, recreational and social activities, and networks between 6 and 12 months, significant change was reported in almost all the clinics apart from BASR and El Wafa (the recreational activities). The reports indicated a worse economic situation and/orless recreational activities post stroke. For the participants as a whole, perception of the themes of work, follow‐up services, and maintenance of networks did not significantly change between the time points 6 and 12 months post stroke (Table 3).

**Table 3:** Differences between 6 and 12 months in patients’ perceptions of their work, financial situation, health insurance, follow‐up, recreational and social activities, and networks

	Sunnaas (*n* = 31)	CRRC (*n* = 35)	Rusk rehab (*n* = 17)	Pol. clin 2 (*n* = 30)	Sheba (*n* = 14)	BASR (30)	El Wafa (*n* = 8)	SU/Högsbo (*n* = 30)	Sichuan Bayi (*n* = 35)
Work	0.09	0.14	0.42	0.01	0.02	0.37	0.73	0.04	0.68
Financial	**0.0001**	**0.0001**	**0.006**	**0.0001**	**0.02**	**0.39**	**0.02**	**0.0001**	**0.0001**
Follow‐up	0.16	0.07	0.16	0.73	NS	NS	NS	0.03	0.53
Recreational	**0.0001**	**0.0001**	**0.002**	**0.0001**	**0.01**	**0.05**	**0.08**	**0.0001**	**0.0001**
Network	0.41	0.03	0.56	NS	0.31	0.56	0.56	0.01	0.32

*p < 0.05.

The qualitative data at 6 and 12 months confirmed the quantitative impression that participants irrespective of country had a maintained physical disability and the participants reported low mood and depression. For some, this had severe consequences with divorce, family problems with quarrels, change of roles, and loss of social position and “respect.” Other physical and psychological disabilities were reported differently between countries. The “western” countries focused more on the psychological and cognitive difficulties post stroke. Fatigue, loneliness, low self‐esteem, anxiety, worries, insecurity, and increased sensitivity for sound as well as memory loss and problems with concentration were some examples mentioned making RTW difficult or impossible. However, it also influenced social participation and networks, with decreased spontaneity leading to low physical and social activity.

The Middle East and eastern countries focused more on the physical disabilities and secondary complications like heart problems, falls, and difficulties in mobility reducing or hindering RTW. Gender differences were reported mainly in Middle East countries like Palestine where females, overall, were more reliant on their families in terms of care and financial situation than men were. Men, on the other hand, seemed to be able to RTW in the small family businesses to a certain degree, although not in the leading capacity as before but in a different role. But in the total sample, there were no differences between genders in terms of RTW, as 18.4%/38.5% of the females and 17.5%/39.8% of the males had returned to work at 6 and 12 months post discharge.

Logistic regression models predicted RTW would be 35% and 26% at 6 and 12 months, respectively. The main predictors were younger age, education, and less disability at 6 months and age and country at 12 months (Table 4a and b).

**Table 4:** (a) Logistic Regression model at 6 months predicting 15%–24% of RTW, explanatory factors. (b) Logistic Regression model at 12 months predicting 6%–8% of RTW, explanatory factors

	OR	*p*‐Value	95% CI	
(a)				
Age	1.0	0.009	1.00	1.1
Education	0.91	0.04	0.82	1.0
NIHSS	0.92	0.12	0.84	1.0
mRS	2.7	0.001	1.7	4.3
Country^a^	0.99	0.83	0.86	1.1
(b)				
Age	1.0	0.048	1.00	1.1
Education	0.98	0.61	0.92	1.05
NIHSS	0.96	0.28	0.89	1.0
mRS	1.3	0.24	1.7	4.3
Country^b^	1.1	0.03	1.0	1.3

a Exp(B) in the respective countries ranging from 0.2 to 6.6.

b Exp(B) in the respective countries ranging from 0.6 to 6.6.

When the sample was divided into younger than 60 years and elderly (60 years or older) groups, a difference in RTW emerged. It was mainly persons less than 60 years, 26.7%, who had returned to work at 6 months, whereas only 6.1% of persons over 60 years had done so. There was a slight increase in RTW in both age groups at 12 months with 29.8% returning compared with 8.2% doing so.

### 3.1 Education

Persons who had enjoyed more years of education, lasting more than 11 years, had a higher RTW rate at 6 months compared to those with a lower educational level (less than 10 years), 21.5% and 9.7%. At 12 months post discharge, 24.1% with more education had returned to work versus 12.5% with a lower level of education.

### 3.2 Finance

In total, 50% were able to maintain the same financial situation as before the onset of stroke, whereas the remaining 50% did not. However, there were differences between countries, as low as 29% (Sheba, Israel) up to 87% (El Wafa, Gaza), complaining of a worsened financial situation at 6 and 12 months post discharge. Marital status did not seem to influence the financial situation, 51%/54% of the married, 51%/59% of the single persons and 44%/67% of widowed persons maintained their financial situation at 6 and 12 months. The association between the questionnaire and the LiSat‐11 item financial situation was rho = −0.36 and −0.43, respectively, indicating significant and medium associations (Cohen, 1988).

### 3.3 Follow‐up services

The chance of receiving additional follow‐up services post rehabilitation varied in the different countries from 24% to 100% at 6 months and from 21% to 100% at 12 months. Physical therapy or a combination of disciplines performed in the homes or at an outpatient department were the most common follow‐up services reported.

### 3.4 Recreational activities

At 6 months post discharge from rehabilitation, participation in recreational activities varied from 17% to 80%, and of those, 14%–69% reported their recreational activities to be less than before the stroke.

The association with the LiSat‐11 item recreational activities was significant but small rho = −0.23 and −0.28, respectively (Cohen, 1988).

### 3.5 Networks

Social networks and their maintenance were reported as 29%–86% at 6 months; of these, 19%–83% reported social networks to be less than before the stroke. At 12 months, 16%–69% reported changes in their social networks in 16%–83% of these patients; the social networks were less than before the stroke. The LiSat‐11 item “contact with friends” indicates that participants were rather satisfied/satisfied with this, and the association with the maintenance of networks was significant with rho = 0.19 and 0.42 at 6 and 12 months, respectively, indicating small to medium associations (Cohen, 1988).

## 4 DISCUSSION

Persons included in this study varied even before the onset of stroke in their vocational situation, with the percentage in work ranging from 27% to 86%. At 12 months, RTW was 11%–43%, indicating an even lower employment status. Consequently, many participants reported a reduced financial situation, 10%–70% at 6 months and 10%–80% at 12 months. Postrehabilitation follow‐up services varied in the different countries, with physical therapy as the most common follow‐up service reported. In addition, persons with stroke were less active in recreational activities and experienced reduced networks. Their satisfaction with life as a whole was stable but low at 6 and 12 months post discharge.

Associations between results from the semistructured interviews and related themes in LiSat‐11 were small to moderate at both 6 and 12 months, indicating that they had experienced differences in employment, their financial situations, their recreation, and networks, which only to some degree influenced their perceived life satisfaction. The study shows that education, age, and disability, but also differences between countries, are predictors for RTW.

### 4.1 Work‐vocational situation

The results from this study are in line with other studies reporting a relatively low but varied percentage in RTW after a stroke. A systematic review of RTW in different countries, similar to those investigated in the present study, reported that 19%–73% were able to RTW (Treger et al., 2007). In comparison, persons with stroke from Sweden reported 20% RTW at 1‐year post discharge in the present study: This is in line with a study by Hofgren, Björkdahl, Esbjörnsson, & Sunnerhagen (2007), also from Sweden, reporting 18% RTW at 1 year. In Norway, RWT was 42% at the same time point in the present study. In this study, the severity of the stroke, disability, and ADL at baseline and at discharge post stroke, the fact that the participants were living in urban areas with a higher proportion of highly educated people and more males were similar to that study, so one would have anticipated that RTW would be about the same (Table 2). Sweden and Norway have similar health and social care systems, although recent reforms in Sweden have resulted in different incentive structures. The difference seemed to have consequences on the individual level where participants from Sweden reported low self‐esteem, feelings of less worth to a higher degree than participants did from Norway. However, possible differences in employment rates and perhaps slight differences in social incentives may explain these different tendencies. In addition, differences in stroke related factors, which were not registered in this study, might have influenced the results. Many individuals listed as long‐term sick move from rehabilitation to a pension, rather than reaching the goal of RTW (Treger et al., 2007; Westerlind, Persson, & Sunnerhagen, 2017). The tendencies were the same in this study where patients from six of the nine clinics reported a higher percentage of retirement at 1 year post discharge. However, patients from three of the clinics reported higher levels of unemployment at 1 year post discharge. These differences seem more related to political and administrative regulations than cultural patterns.

A number of barriers may prevent labor market participation by disabled pensioners and others with a reduced ability to work. A common explanation of the low employment rates in these groups are the attitudes and the willingness of employers to hire individuals with reduced ability to work. Such attitudes will affect the possibilities of getting a job, and therefore, the potential effects of a reform designed to change the behavior of disabled pensioners (Bråthen & Nielsen, 2016).

In addition, the incentives to work may be so small that participants with disability may lose support because they work so little or because travel expenses exceed the earnings of part time work (Treger et al., 2007). Suggestions for increasing the ability to work among disabled persons are flexible working hours, adaptations of the workplace, education, and increased competence (Bråthen & Nielsen, 2016).

The KOSCO study from Korea reported that 60% returned to work at 6 months in a population of stroke victims with minor disabilities (Chang et al., 2016). Bonner et al. (2016) reported that 52.5% returned to work after 3 months to 2 years post stroke in a relatively young group (<60 years old), with mild to moderate stroke (mRS ≤ 3) in India. In comparison, 17% in the urban area and 11% in the rural area return to work in China at 1 year post discharge. The present study included persons with severe stroke (NIHSS total ranging from 6.9 to 9.9 at baseline), which in itself is a negative predictor for RTW (Lindström, Röding, & Sundelin, 2009). This may explain the lower RTW numbers among these participants. However, the results also visualize the success of specialized rehabilitation for this group of severely disabled persons with stroke, who in many ways were less likely to RTW. Subgroup analysis in this respect may be crucial in individualizing and customizing vocational rehabilitation programs for persons with stroke.

Consequently, the financial situation for all persons with stroke involved in the study (regardless of country) was affected. However, the size of the impact varied, indicating to what extent social security nets existed in the form of private or public insurance, and to what extent these covered daily expenses. For example, there was a difference between the centers in the urban and rural areas of China, reflecting different local systems of health insurance, which had consequences for individuals. Another explanatory variable for a reduced financial situation may be the shift from working status to unemployed or retired. People with low socioeconomic status, who live alone and have worse health, are more likely to receive a disability pension compared with people with high socioeconomic status (Treger et al., 2007).

The loss of a work role may further influence and possibly decrease social networks and reduce life satisfaction (Daniel et al., 2009; Vestling et al., 2003; Wolfenden & Grace, 2015). This tendency was also present in this study where the social networks were reported to be reduced 20%–70% at 6–12 months post discharge. The clinics in China, United States, Palestine, and Sweden reported a higher negative change in social networks than clinics in Norway and Israel, indicating possible cultural differences.

### 4.2 Recreational and follow‐up services

Vocational status may also take account of recreational activities, which can be seen as replacements for working activities, maintaining social interactions, social networks, and perceived life satisfaction.

There was a diversity in reports of participation in recreational activities post stroke, with reports decreasing between 6 and 12 months post discharge. Moreover, even though many appeared to be active in recreational activities, when asked to compare these with prestroke activities, many reported a decrease in levels, again here accentuated at the longer time post discharge.

Persons with stroke from the clinics Sunnaas, CRRC, and Bayi reported highest levels of recreational activities, possibly explained by cultural differences. In Norway, there is a high level of participation in recreational activities and outdoor activities among the general population; in China, activities like dancing, Tai‐Chi, and social gatherings are popular in parks and other settings, which may partly explain the higher degree of activities in these countries.

Participants received many follow‐up services at 6 and 12 months. However, there was a difference for follow‐up ranging from as low as 20% up to 100%. The difference may partly be explained by the infrastructure of services in the different countries. Those countries with less follow‐up were the ones where community‐based services were less developed. To what extent these follow‐up services are important for RTW remains unclear, as well as to what extent they were tailored to the specific needs of the individuals (Martinsen, Kirkevold, & Sveen, 2015). No associations between follow‐up services and RTW could be established in the present study, nor were any explanatory factors discerned. However, the follow‐up services reported here were related to issues of health and impairments and mainly related to motor function, not vocational training as such, which may explain the low associations with RTW (Baldwin & Brusco, 2011).

In summary, the novelty is that these are data from several clinics at the same time, with same global environment when it comes to development. The data show individuals who live under different circumstances and perceive their lives equally dissatisfying. They have different possibilities of getting back to work, depending on differences more related to political and administrative regulations than cultural patterns, health policies, possibilities to receive rehabilitation and training post discharge from specialized rehabilitation. This is especially apparent when looking at the two assumingly similar countries Norway and Sweden where differences in policies regarding work legislation seem to lead to severe consequences for the individual in terms of RTW and their financial situation.

### 4.3 Limitations

Any generalization of interventions or models from this cohort sample must be done with caution, because of the small samples collected. In addition, this study does not represent an unselected stroke sample; admission to the participating clinics was the main inclusion criterion in addition to stroke. Moreover, knowledge about the patients participating in this study is limited as, for example, there had been no assessment of cognitive status or fatigue, which are known to be important for RTW after stroke (Andersen, Christensen, Kirkevold, & Johnsen, 2012; Malterud, 2012; Treger et al., 2007).

However, the present results may serve as indicators from a unique multicultural sample of severely disabled persons with stroke giving narrative descriptions of RTW, associations with perceived life satisfaction and related factors.

In future, more in‐depth studies are warranted to establish possible interventions to improve vocational rehabilitation in this subgroup of persons with stroke.

## 5 CONCLUSION

The study shows that multicomorbidity prestroke will be detrimental for RTW after stroke. In addition, persons with severe stroke are more likely not to return to work because of disability. Furthermore, education, age, and post stroke disability predict the level of RTW. In addition, differences between countries influence RTW for persons with stroke. Persons with stroke received a varied extent and form of follow‐up, were less active in recreational activities, and experienced reduced social networks. Their satisfaction with “life as a whole” was low at 6 and 12 months post discharge. The associations between related items in LiSat‐11 and the results at 6 and 12 months were small to moderate, indicating that the differences in vocation, financial situation, recreational, and networks that they experienced mattered only to a minor degree in their perceived life satisfaction. Return to work after severe stroke and specialized/comprehensive rehabilitation is possible, mainly depending on the extent of the disability, age, and education as well as social construct. Altered financial situation, reduced social networks, and reduced satisfaction with life are common psychosocial situations for these patients.

## CONFLICTS OF INTEREST

None declared.
